# SARS-CoV-2 infection in domestic and feral cats: current evidence and implications

**DOI:** 10.1080/01652176.2021.1962576

**Published:** 2021-08-18

**Authors:** Khan Sharun, AbdulRahman A. Saied, Ruchi Tiwari, Kuldeep Dhama

**Affiliations:** a Division of Surgery, ICAR-Indian Veterinary Research Institute, Izatnagar, India; b Department of Food Establishments Licensing (Aswan Branch), National Food Safety Authority (NFSA), Aswan, Egypt; c Ministry of Tourism and Antiquities, Touristic Activities and Interior Offices Sector (Aswan Office), Aswan, Egypt; d Department of Veterinary Microbiology and Immunology, College of Veterinary Sciences, UP Pandit Deen Dayal Upadhayay Pashu Chikitsa Vigyan Vishwavidyalay Evum Go-Anusandhan Sansthan (DUVASU), Mathura, India; e Division of Pathology, ICAR-Indian Veterinary Research Institute, Izatnagar, India

**Keywords:** COVID-19, SARS-CoV-2, feral cat, mink, domestic cat, interspecies transmission

## Abstract

Current evidence indicates that cats play a limited role in COVID-19 epidemiology, and pets are probably dead-end hosts of SARS-CoV-2 and pose negligible risks of transmission to humans. Still, one health concept is to be adopted widely as a component of mitigation strategies to tackle the ongoing pandemic. Therefore, in terms of the magnitude of infection and potential to transmit SARS-CoV-2 to humans, our surveillance efforts should mainly focus on mustelids (especially minks, ferrets, and others) for early detection and control of infection. This will ensure that SARS-CoV-2 will not get established in the wild animal population of these susceptible species. We agree with Dr. Passarella Teixeira on the possibility of domestic and feral cats acting as an urban reservoir, subsequently transmitting the virus to human beings. However, it is less likely that such a phenomenon will be reported even if it has occurred due to the efficient and extensive human-to-human transmission of SARS-CoV-2.

We would like to thank Dr. Passarella Teixeira at Tropical Medicine Center, University of Brasília, for her interest in our paper (Sharun et al. 2021a) and for taking the time to express her views (Passarella Teixeira 2021). The susceptibility of cats to severe acute respiratory syndrome coronavirus 2 (SARS-CoV-​2) did not come as a surprise mainly because of the fact that they were previously reported to be infected with severe acute respiratory syndrome coronavirus (SARS-CoV) during the 2003 outbreak (Rodriguez-Morales et al. 2020). The susceptibility of cats (*Felis domesticus*) to SARS-CoV infection was confirmed by experimental studies (Martina et al. 2003). The infected cats were also found to efficiently transmit SARS-CoV to previously uninfected animals that are housed with them (Martina et al. 2003). However, the researchers were not able to confirm cat-to-cat and cat-to-human transmission in natural conditions. Considering this previous history with zoonotic coronavirus, felids, including domestic cats, were widely studied for their ability to transmit SARS-CoV-​2 to susceptible cats and humans. In addition to that, apart from reports of SARS-CoV-2 infection in dogs, cats, gorilla, and minks, captive felids such as tiger (*Panthera tigris*), lion (*Panthera leo*), puma (*Puma concolor*), and snow leopard (*Panthera uncia*) were also found to be susceptible to SARS-CoV-2 infection under natural conditions (Dhama et al. 2020; Tiwari et al. 2020; Delahay et al. 2021; Sharun et al. 2021a). The majority of these cases were linked to human-to-animal transmission of SARS-CoV-2 from asymptomatic zoo workers and caretakers (Sharun et al. 2021a).

SARS-CoV-2 has been found to replicate in domestic cats, and cat-to-cat viral transmission has been observed under experimental conditions (Shi et al. 2020). Reports from different countries have confirmed human-to-cat SARS-CoV-2 transmission as an anthropogenic route of infection from COVID-19-positive pet owners. The infected cats exhibited mild to severe forms of the disease (Halfmann et al. 2020; Decaro et al. 2021; Maurin et al. 2021; Hosie et al. 2021a, 2021b; Sharun et al. 2021a). The binding affinity between SARS-CoV-2-receptor-binding domain (RBD) and the angiotensin-converting enzyme 2 (ACE2) receptor of cats was high compared to that in bats (Ma et al. 2021). However, the transmission of the virus from cat to human has not been reported yet under natural circumstances. One recent study from Switzerland demonstrated SARS-CoV-2 RNA from the cats' fur and bedding, suggesting the feasibility of mechanical transmission. It also emphasizes the importance of appropriate hygienic and sanitation measures to be followed by pet owners to avoid infection (Klaus et al. 2021). Serological evidence of SARS-CoV-2 exposure to stray cats has also been very recently documented in Spain, warranting the need for large-scale epidemiological studies to identify the role played by cats in the ongoing pandemic (Villanueva-Saz et al. 2021).

Coronavirus-derived and cell receptor angiotensin-converting enzyme RNA/proteins have been detected in the midgut of domestic cat flea (*Ctenocephalides felis*), indicating a probability that such ectoparasites may play the role of reservoirs as well as biological and/or mechanical vectors of SARS-CoV and related betacoronaviruses (Villar et al. 2020). Similar studies should be conducted to investigate the possible role of the cat flea in disseminating SARS-CoV-2. Additionally, the predation of bats by domestic cats and the close contact between these two species might also contribute to the transmission of SARS-CoV-2 to bats. Although this is a hypothesis, the possibility for such a scenario has to be studied further by assessing the risk and developing adequate prevention and control strategies to counter it using one health approach (Salinas-Ramos et al. 2021).

However, when we compare the magnitude of SARS-CoV-2 infection in domestic cats and other felids to that of COVID-19 in humans (human-to-human transmission), the reports of SARS-CoV-2 spillover to felids is very small and can only be characterized as sporadic. On the contrary, infected minks were able to rapidly transmit the virus to other uninfected minks and human beings, causing a great panic among the mink farms of Europe (Sharun et al. 2021b). Between April and November 2020, 69 out of 127 mink farms in the Netherlands were infected with SARS-CoV-2, triggering large-scale culling to prevent further spread of the virus (van Aart et al. 2021). The continuous and rapid transmission of SARS-CoV-2 in the mink population also contributed to the emergence of a mink-associated SARS-CoV-2 variant that was later identified in human beings confirming mink-to-human transmission (Sharun et al. 2021b).

Recently, feral cats living in close proximity to infected mink farms were found to be infected with SARS-CoV-2 due to mink-to-cat transmission (van Aart et al. 2021; Sharun et al. 2021a). This is the first report that confirms interspecies transmission of SARS-CoV-2. Furthermore, in the scenario where there is no cat-to-cat transmission, there is a 12% (10%-18%) chance that cats can get infected by mink (van Aart et al. 2021). In a serological survey conducted in the city of Zaragoza (Spain), immunosuppressed stray cats (due to concomitant infections with *Toxoplasma gondii*, *Leishmania infantum*, and feline immunodeficiency virus) were found to be more susceptible to SARS-CoV-2 infection (Villanueva-Saz et al. 2021). Though experimental and natural infections have confirmed the susceptibility of domestic cats to SARS-CoV-2, serial passaging of the virus between the cats attenuated the viral transmissibility significantly (Bao et al. 2021). Therefore, cat-to-cat transmissibility of SARS-CoV-2 can get reduced over time compared to the sustained human-to-human transmission. This attenuation can be attributed to the amino acid variations in the receptor-binding domain (RBD) sites of angiotensin-converting enzyme 2 (ACE-2) receptor of cats compared to humans (Bao et al. 2021). Although cats can get infected with SARS-CoV-2 from human beings (Sharun et al. 2020; Hosie et al. 2021a), the possibility of cat-to-cat (under natural circumstances) and cat-to-human transmission is less likely to occur as they lack the capacity to facilitate the onward transmission of the virus. Therefore, extensive epidemiological investigations should be conducted among the cats living in geographic areas with a high prevalence of SARS-CoV-2 in humans to confirm the role played by domestic cats in the ongoing pandemic (Decaro et al. 2021).

According to Totton et al. (2021), the following two essential criteria must be met to demonstrate the cat-to-human transmission of SARS-CoV-2:

1. The individual should undergo an effective quarantine period, followed by negative PCR and serologic testing that eliminates the potential for undetected SARS-CoV-2 infection.
2. The individual must remain isolated from all other sources of SARS-CoV-2 from the start of the effective quarantine period, through exposure to the infected cat, development of symptoms, and diagnosis.

Despite limited evidence of cat-to-cat SARS-CoV-2 transmission (only in experimental conditions) and lack of evidence on cat-to-human transmission, we should not forget the possible role that could have played by the domestic cats in disease dissemination. Therefore, further investigations should focus on surveillance and monitoring of cats with detailed molecular and seroepidemiological studies, as well as adopting adequate prevention and control strategies for limiting zoonotic transmission from cats to human beings. This can be further strengthened by adopting one health approach (Bessière et al. 2021; Braun et al. 2021; Chaintoutis et al. 2021; Davis and Innes 2021; do Vale et al. 2021; Sharun et al. 2021a; Zhao et al. 2021). Individuals affected with COVID-19 should avoid close contact with their pet cats and must follow appropriate hygienic and sanitation measures to prevent the risk of SARS-CoV-2 infection. Furthermore, the recommended quarantine measures have to be extended to cats living in a COVID-19 positive household environment (Calvet et al. 2021; Pagani et al. 2021). Current evidence indicates that cats play a limited role in COVID-19 epidemiology, and pets are probably dead-end hosts of SARS-CoV-2 and pose negligible risks of transmission to humans. Still, one health concept is to be adopted widely as a component of mitigation strategies to tackle the ongoing pandemic.

Therefore, in terms of the magnitude of infection and potential to transmit SARS-CoV-2 to humans, our surveillance efforts should mainly focus on mustelids (especially minks, ferrets, and others) for early detection and control of infection. This will ensure that SARS-CoV-2 will not get established in the wild animal population of these susceptible species. We agree with Dr. Passarella Teixeira on the possibility of domestic and feral cats acting as an urban reservoir, subsequently transmitting the virus to human beings (Passarella Teixeira 2021). However, it is less likely that such a phenomenon will be reported even if it has occurred due to the efficient and extensive human-to-human transmission of SARS-CoV-2.
